# Development of a simple test to quantify the consolidation properties of liquefied granular materials

**DOI:** 10.1007/s11440-024-02434-5

**Published:** 2024-11-18

**Authors:** L. Tauskela, O. Adamidis, W. A. Take

**Affiliations:** 1 Queen’s University, Kingston, ON K7L 3N6 Canada; 2 Department of Engineering Science, University of Oxford, Oxford, OX1 3PJ UK

**Keywords:** Consolidation, Debris flow, Liquefaction, Pore pressure, Physical modelling

## Abstract

Liquefaction can have devastating consequences by causing increased mobility of debris flows, tailings dam breaches, and settlement following seismic shaking. Observations on the consolidation behaviour of liquefied soils in 1-g or centrifuge shake table tests have permitted significant advancements in analytical and numerical methods to predict the rate and magnitude of consolidation settlement. However, advanced consolidation models introduce material parameters which are currently difficult to define quickly and at low cost. The objective of this paper is to demonstrate the development of a simple, low-cost test that can be used to quickly estimate the spatially and temporally varying coefficient of consolidation, which controls post-liquefaction settlements. Using the proposed simple setup, experiments were conducted on four uniformly graded sands of varying grain size and on one well-graded mixture. A range of analytical and numerical approaches from literature were assessed for their ability to back-analyse the observed pore pressure dissipation and settlement. Estimated values for the coefficient of consolidation were comparable between models and estimates of surface settlement matched well the experimental results. The simplicity of the proposed test combined with its low-cost and mobile nature, raise significant possibilities for quick estimations of the evolution of the coefficient of consolidation post-liquefaction.

## Introduction

Liquefaction is the sudden loss of strength and stiffness caused by rapid loading of loose, saturated granular materials. Liquefaction can have widespread implications for infrastructure and human safety. It has been shown to increase the mobility of debris flows (e.g. [4, 16, 18, 22]), to contribute to tailings dam breaches (e.g. [6, 7, 17]), and to drive infrastructure to the ultimate or serviceability limit state following seismic shaking (e.g. [11, 20, 21]).

Liquefaction can occur when a triggering event rapidly loads a loose, saturated granular soil which has the tendency to contract upon shearing. Possible triggers include earthquakes, the initiation of a landslide in a debris flow catchment, or a tailings dam breach. If shearing occurs too rapidly for water to escape through the pore spaces, pore water pressures greater than hydrostatic, termed excess pore pressures, can develop. Excess pore pressures decrease grain contact forces, causing effective stress to drop, and can lead to liquefaction.

Consolidation is the process following liquefaction when excess pore pressures dissipate back to hydrostatic, grain contact forces are regained, and additional settlement occurs. The literature on the dissipation of excess pore pressure is built on the one-dimensional consolidation equation, a form of the diffusion equation, proposed by Terzaghi [19] and shown in Eq. 1.

1 $$\begin{array}{*{20}c} {\frac{{\delta \overline{u}}}{\delta t} = c_{v} \frac{{\delta^{2} \overline{u}}}{{\delta z^{2} }} 1} \\ \end{array}$$

where *c*_*v*_ is the coefficient of consolidation, $\overline{u}$ is excess pore pressure, *t* is time, and *z* is elevation. In Terzaghi’s equation, *c*_*v*_ is taken as a constant.

Estimation of *c*_*v*_ from experimental results requires a mechanism to liquefy the soil, high quality pore pressure measurements, and analytical or numerical methods to back-analyse the observed pore pressure response. Settlement measurements can add to the reliability of estimated *c*_*v*_ values. Liquefaction is most commonly triggered experimentally using a shake table under either 1 g conditions (e.g. [9]) or under enhanced gravity in a geotechnical centrifuge (e.g. [1, 3]). At the laboratory bench scale, fluidisation experiments using an upward hydraulic gradient have been successfully used to determine the coefficient of consolidation as well as variations of stiffness and permeability with effective stress [10]. Using a somewhat less controlled approach, Major and Washington [15] incrementally poured pre-liquefied material into a small tank to observe the behaviour of liquefied soils at low effective stresses. A shortcoming of this method, which requires the incremental addition of material, is that it is difficult to measure dissipation and settlement when the consolidation time is lower than the time required to add each layer.

Experimental observations of post-liquefaction consolidation inspired analytical and numerical back-analysis methods. Such methods aim to fit experimental results either using Fourier series approximations [3, 15], or numerical solutions of the consolidation equation [1]. As models have advanced, more parameters have been added to increase their accuracy and capabilities. While Major and Washington, [15] and Brennan and Madabhushi, [3] use an average time-dependent *c*_*v*_ across a layer, the model of Adamidis and Madabhushi [1] calculates *c*_*v*_ as a function of effective stress, by defining permeability and one-dimensional stiffness. It hypothesises that the one-dimensional stiffness of liquefied soil is non-zero and includes a transition zone from the low effective stress stiffness to a power law at large effective stress. To realise this, 7 empirical parameters are needed, which include the effective stress boundaries of the stiffness transition zone. The process requires additional effort to fit experimental data but it has the ability to model the advancement of the boundary between liquefied and solidified parts (solidification front), it produces a temporally and spatially variable consolidation coefficient, and it can predict post-liquefaction settlement. A justifiable criticism of advanced models, such as Adamidis and Madabhushi [1], is whether these additional material parameters can be defined at sufficiently low cost and timeliness, potentially limiting adoption of the method in practice. While hydraulic conductivity testing and one-dimensional stiffness at effective stresses greater than 5 kPa are easily obtainable through standardised testing, their nonlinear variation at low stresses is currently obtained only by back-analysis of shake table or centrifuge testing.

The objective of this paper is to propose a low-cost, rapid test through which such consolidation parameters can be easily estimated, focusing on a variable coefficient of consolidation that can predict post-liquefaction settlement. A test is proposed for saturated granular materials, that are placed in a cylinder and rapidly liquefied through an impulse load. Post-liquefaction excess pore water pressures and surface settlement are measured and analytical and numerical methods proposed in literature are used to back-calculate consolidation properties. In the remainder of this paper, we describe the experimental setup and procedure, the application and applicability of the method, and the back-calculation of parameters.

## Materials and methods

### Experimental setup and instrumentation

In considering the design of a simple experimental apparatus to quantify consolidation parameters, we make three observations:

1. We are only interested in the post-liquefaction consolidation response of the soil. Liquefaction should be initiated rapidly so that changes in density of the material prior to consolidation are limited. The details of the dynamic signal to initiate liquefaction are not of particular interest for our post-liquefaction analysis.
2. The parameter that controls post-liquefaction consolidation is the coefficient of consolidation, *c*_*v*_. Sufficient evidence in literature shows that post-liquefaction *c*_*v*_ cannot be taken as a constant (e.g. [1, 3]). The most elusive aspects of response, related to solidification of liquefied soil and settlement generation, are controlled by the value of *c*_*v*_ at low effective stresses. Given this, 1 g testing is preferable, as effective stresses remain low.
3. The thickness of the liquefied layer must be sufficient to capture the advancement of the solidification front for the range of grain sizes and permeabilities of interest, as we show that this is a useful way to estimate the minimum value of *c*_*v*_.

**Fig. 1:**
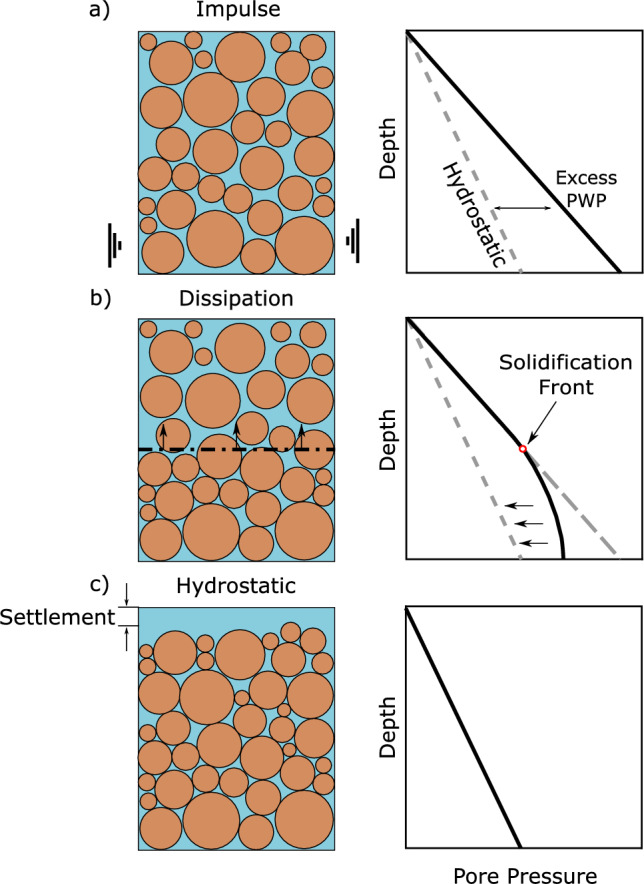
**a** An impulse causes immediate generation of excess pore pressures, minimising grain contacts and reducing effective stress; **b** The solidification front begins at the base of the layer and travels upwards. Upon the arrival of the solidification front, consolidation begins as excess pore pressures start to dissipate to hydrostatic levels; **c** Following consolidation, excess pore pressures have dissipated and settlement has occurred due to grain rearrangement

In the simple test we propose, we use a single impulse delivered at the base of a 500 mm high cylinder of loose saturated material under 1 g conditions. The cylinder is instrumented with a vertical array of pore water pressure transducers in fixed positions and a displacement gauge to monitor surface settlement. An illustration of the proposed test, including the expected pore pressure profiles following the impulse, during dissipation, and following dissipation, is shown in Fig. 1. In practice, the method of delivering this impulse can be as simple as a kick from a steel-toed safety shoe to the side of the container to serve as a field-deployable impulse of very short-duration that generates both shear and pressure waves. It is important to reiterate that the aim of the impulse is not to reproduce a realistic seismic motion, but rather to quickly liquefy the soil so that the post-liquefaction response can be monitored. The monitored part of response corresponds to the post-earthquake reconsolidation in a deposit.

This testing method is a very simple analogue to the well documented field of liquefaction analysis using controlled blasting (e.g. [8]). The excess pore pressures typically produced through blasting are considered too low to cause compression-induced slippage between grains, thus no change to the soil fabric should be expected from compression. Given that the transient excess pore pressures generated during the impulses in the setup proposed here were significantly lower than those generated through blasting, no compression-induced fabric change should be expected. Upon unloading of the passing P-waves generated through blasting, near-field S-waves are produced, which can induce slippage between the grains and therefore changes to the soil fabric. Jana and Stuedlein [14] used velocity measurements post-blasting within a deposit of sand to show that it is post-P-wave, SV-waves that cause shear strains and the accumulation of excess pore water pressure, as suggested by Gohl et al. (2001). A similar process is expected to take place in the proposed setup, with shear-induced, ‘residual’ excess pore pressures at the end of the impulse, when the back-calculation analysis begins.

Another point worth consideration is the link between maximum shear strain and post-liquefaction volumetric strain established through element tests (e.g. [12]). In a simple soil column test as the one proposed here, the magnitude and distribution of maximum shear strain with depth is difficult to capture. However, drawing an analogue to blasting-induced liquefaction, the shear strains developed should be expected to be just enough to cause liquefaction (e.g. [14]). Indeed, this is a reasonable assumption for most level soil deposits that experience full liquefaction, since once the required strain to trigger liquefaction is reached, the liquefied soil is unable to transmit the shear stresses required to reach higher shear strains. For instance, Ishihara and Yoshimine [12], when applying their element test-based method to soil-columns, barely ever calculated shear strains higher than the threshold for triggering liquefaction. Inherent in our analysis is the assumption that for our level soil column setup, the shear strain is not able to reach values higher than those required for liquefaction triggering. This assumption allows us to use the same soil compressibility low throughout the liquefied depth of the soil column. Shear strains below the threshold for liquefaction triggering can be attained during a test, but in such cases the expected post-test volumetric strain can be related to the maximum excess pore pressure (e.g. [5]), an effect which can be adequately captured through the initial pore pressure distribution, without an updated compressibility definition.

**Fig. 2:**
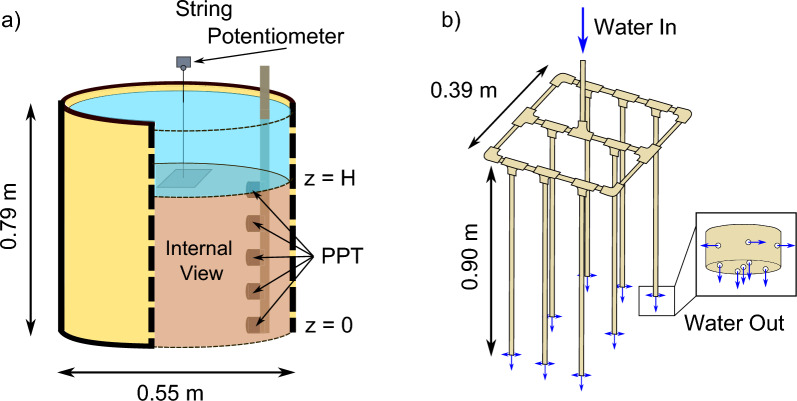
**a** A 0.79 m × 0.55 m cylindrical container held saturated granular material. Pore pressure transducers were affixed to a vertical pole along the sidewall. One PPT rested at the base, z = 0, and another PPT rested on the material surface, z = H. A string potentiometer affixed to a metal plate was placed on the material surface and captured surface settlement; **b** A liquefying device was used to ensure loose placement of the granular material. Water flowed in from a 2″ water main and split into 8 vertical ¾” PVC pipes that were fitted with caps with 3 mm holes to increase effluent water pressure

Though all experiments presented in this paper were performed in a lab setting, the method was developed so that it could in the future become field-deployable. The proposed test is better suited to uniform grain size materials, to limit the risk of grain segregation during sample preparation. To create uniformly loose poorly graded specimens we developed a liquefying device (liquefier) while for larger grain sizes we used submerged placement. The liquefier is presented in Fig. 2b. The liquefier was constructed from ¾” diameter PVC tubing. It consisted of eight 0.8 m long vertical arms arranged in a square grid measuring 0.39 m by 0.39 m. Water flowed in from a 2″ water main and was stepped down before entering through the centre of the liquefier and split to the vertical arms. The ends of the arms were capped with PVC caps that had five 3 mm holes drilled into their base in an ‘X’ formation and four 3 mm holes equally spaced around the perimeter. At the beginning of each test, the liquefier was held above the surface of the material and the water turned on. The arms of the liquefier were then inserted vertically into the material, fluidising the soil around them. The liquefier was subsequently moved laterally to ensure fluidisation of the whole cylinder. The liquefier was finally removed, leaving the material in a uniform, loose state.

Though the proposed test is more easily applicable for poorly graded materials, materials in the field are often well graded. To test whether the proposed test might be appropriate for well-graded materials while remaining potentially field-deployable, such a material was prepared using submerged placement. It should be noted that all materials examined, including the well-graded one, had no fines content, which would make submerged placement prohibitive. During specimen preparation, a height of water of approximately 0.3 m above the soil surface was kept. Material was slowly introduced and placed under the water surface with a minimum drop height, to avoid segregation.

After the material layer was created, the plate of the SP was placed on the surface and the impulse load was applied. Generated excess pore pressures were captured by the PPTs and the surface settlement was captured by the SP.

### Material

**Fig. 3:**
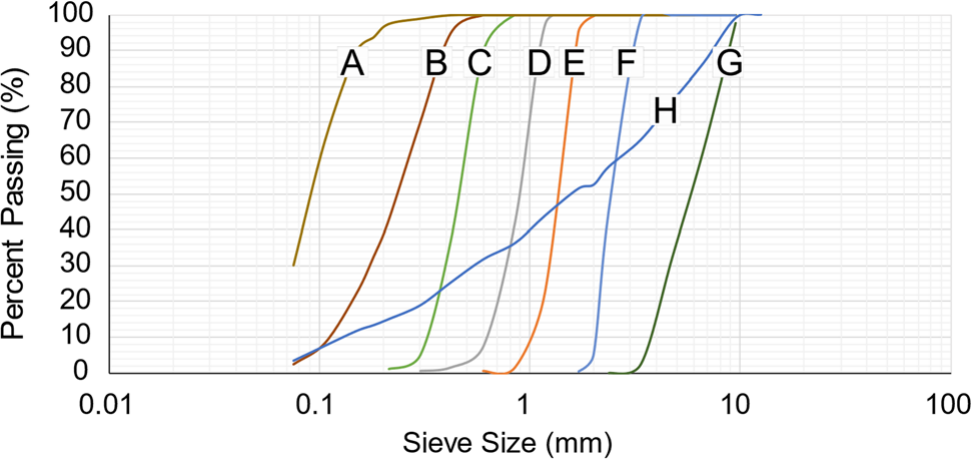
Grain size distribution curves of seven uniform materials, labelled A to G, and one well-graded mixture, H, which was created from the seven uniform materials and designed to mimic real debris flow mixtures

Seven uniform granular materials were used, labelled A to G. A well-graded mixture was also created, labelled H, comprising a mixture of materials A to G, to assess the applicability of the method. The grain size distribution of material H produces high runout in small-scale flume testing and was thus chosen to represent natural debris flows [2]. The grain size distributions of the eight materials are presented in Fig. 3.

The permeabilities of materials A, B, C, D, and H were determined from constant head permeameter tests using a rigid wall permeameter. ASTM standard D2434-22 was followed with some modifications. A permeameter with an inner diameter of 6.36 cm and height of 13 cm was used. The sample height under investigation was 6.36 cm. Material was placed saturated and not compacted to simulate test conditions. Deaired deionised water flowed through the system prior to testing. Material A was left overnight to saturate, while the remaining coarser materials were left for at least one hour. Three flow measurements were taken at three hydraulic gradient levels and the hydraulic conductivity was averaged.

The hydraulic conductivity of materials E, F, and G was too high to be determined using the method outlined in this paper. A summary of material gradation, hydraulic conductivity, and method applicability, given the dissipation time for a layer 30–50 cm thick, is presented in Table 1. Replicate tests were conducted for each of the five materials. A summary of the sensor location and test details is presented in Table 2.

**Table 1:** Grain size, permeability, and method applicability of the uniform and well-graded materials

Material label	Gradation	D_10_ (mm)	D_50_ (mm)	Permeability		Method applicability	
				k (m/s)	e	Dissipation Time(s)	Y or N
A	Uniformly Graded	< 0.075	0.09	9.24 × 10^–5^	0.85	100–150	Y
B		0.11	0.23	2.79 × 10^–4^	0.75	20–30	Y
C		0.316	0.46	8.02 × 10^–4^	0.68	5–10	Y
D		0.615	0.89	1.16 × 10^–2^	0.69	0.5–1	Y
E		0.971	1.36	–	–	< 0.5	N
F		2.04	2.44	–	–	~ 0	N
G		3.66	5.81	–	–	~ 0	N
H	Well Graded	0.131	1.59	1.57 × 10^–4^	0.36	5–10	Y

**Table 2:** Summary of PPT locations and elevations for replicate tests of each material

Material label	Number of PPTs	Height of material (m)	PPT elevations (m)
A	7	0.39	0, 0.03 0.10, 0.16, 0.23, 0.32, 0.39
B	7	0.40	0, 0.03, 0.10, 0.15, 0.22, 0.30, 0.40
C	6	0.47	0, 0.13, 0.19, 0.26, 0.37, 0.47
D	6	0.44	0, 0.09, 0.16, 0.25, 0.35, 0.44
H	5	0.31	0, 0.12, 0.19, 0.24, 0.31

## Pore pressure response to impulse

**Fig. 4:**
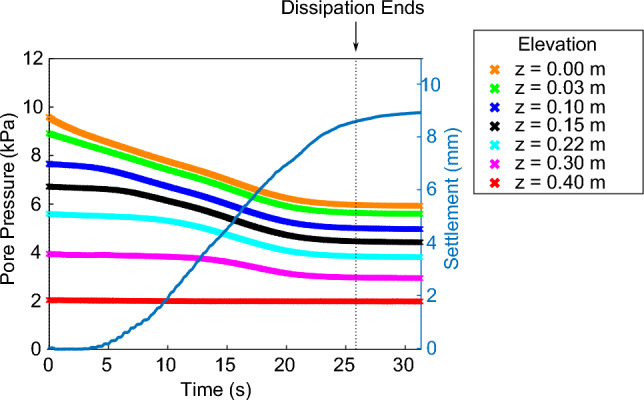
Typical pore pressure dissipation results of material B. Pore pressure conditions prior to the impulse were hydrostatic at each of the seven PPTs located at various elevations from base to surface. Initial pore pressure at the surface was not zero due to ponding water. Dissipation and settlement began immediately following the impulse. Dissipation ended when pore pressure conditions returned to hydrostatic

An impulse applied at the base of the loose, saturated granular materials was sufficient to cause liquefaction and subsequent consolidation of the granular layer. Initially, the pore pressure profile was hydrostatic. Upon impulse, pore pressures rapidly increased. Following the impulse, pore pressures dissipated back to hydrostatic. The surface settled as the excess pore pressures dissipated, with approximately 5 s delay. Typical results for material B are presented in Fig. 4.

**Fig. 5:**
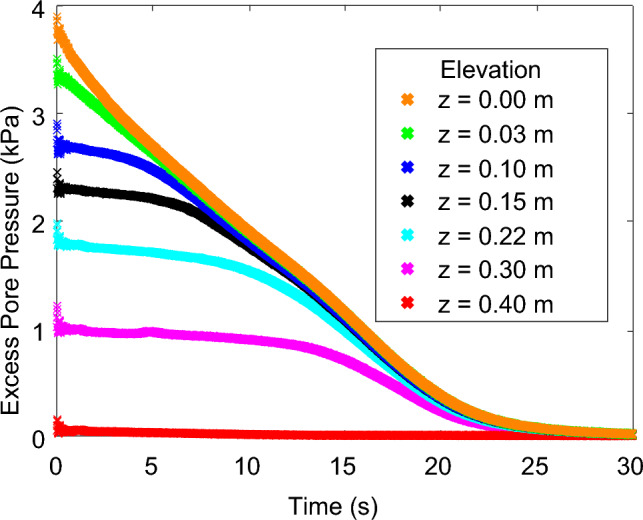
Excess pore pressure dissipation time histories for material B. Dissipation began at the base, z = 0. Dissipation at higher elevations began after the arrival of the upwards moving solidification front. Dissipation at all elevations ended at the same time

Excess pore pressures were calculated as the magnitude of pore pressures in exceedance of hydrostatic. Upon initial liquefaction, excess pore pressure took the values of pre-liquefaction vertical effective stress. Afterwards, excess pore pressures gradually dissipated. Excess pore pressure dissipation in a 0.40 m thick sample of material B is presented in Fig. 5. The boundary between fully liquefied and solidified, consolidating soil is called the “solidification front”. The solidification front travelled from the base to the top of the container. Before its arrival, excess pore water pressures remained elevated as the soil was liquefied. After its arrival, excess pore pressures gradually dissipated as the soil consolidated.

**Fig. 6:**
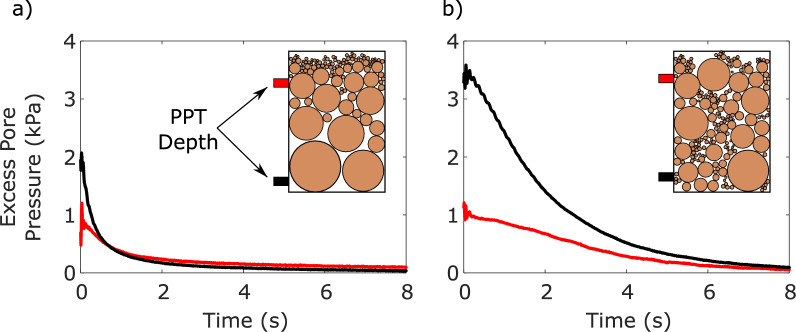
**a** Excess pore pressure dissipation of a material H sample that segregated following use of the liquefier. Finer materials rose to the surface while gravel remained at the bottom, preventing liquefier insertion, and limited excess pore pressure development at the base, which dissipated quickly through the gravel layer; **b** A sample of material H hand placed by sprinkling material into the container to ensure loose placement. Minimal segregation allowed development of excess pore pressures throughout

The importance of avoiding segregation for well-graded materials is outlined in Fig. 6. Figure 6a presents results from a specimen prepared using the liquefier, which caused grain segregation. The coarse grained lower part of the specimen did not fully liquefy after the impulse and its increased hydraulic conductivity led to rapid dissipation of excess pore pressures. The fine grained upper part of the specimen did liquefy but excess pore pressures started dissipating quickly after the impact, since the initial position of the solidification front was close to the surface of the specimen. These results cannot be considered representative of an actual well-graded material. In contrast, submerged placement led to a representative specimen, that fully liquefied and exhibited the expected behaviour of advancement of the solidification front followed by excess pore pressure dissipation during consolidation. The impulse-nature of the imposed loading leads to fluidisation conditions of very short-duration that limit segregation during testing and thus allow interpretation under the assumption of a continuum. The relevant results are shown in Fig. 6b.

The applied impulse was sufficient to cause full liquefaction for almost all of the tested materials. This was assessed by calculating the excess pore water pressure ratio, *r*_*u*_, at the base of the container. The excess pore water pressure ratio is the ratio of excess pore water pressure over initial vertical effective stress. The definition is given in Eq. 2:

2 $$\begin{array}{*{20}c} {r_{u} = \frac{{\overline{u}}}{{\sigma^{\prime }_{v,0} }}} \\ \end{array}$$

where $\overline{u}$ is excess pore water pressure and $\sigma^{\prime }_{v,0}$ is the initial vertical effective stress.

when $r_{u}$ takes a value equal to unity then full liquefaction occurs.

**Fig. 7:**
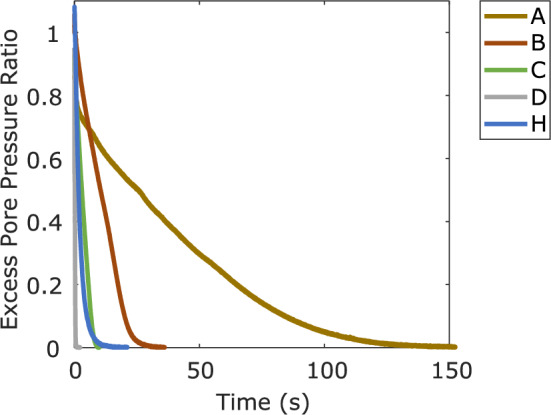
Dissipation time for materials A, B, C, D, and H, in terms of excess pore pressure ratio at the base to demonstrate time scale variability

Samples B, C, D, and H had excess pore pressure ratios close to 1 immediately following the impulse, as shown by Fig. 7. The most fine grained sample, A, reached a high value of $r_{u}$ but not quite unity. The dissipation time for each material to hydrostatic conditions varied widely. Full excess pore pressure dissipation took approximately 125 s for Material A while less than a second was needed for material D. The wide variation in the rate and time required for dissipation reflects the range of consolidation parameters of the tested materials. For more coarsely grained materials (E, F, G) excess pore pressures dissipated too rapidly for the results to be used. This indicates that the proposed test with the applied instantaneous impulse is better suited for uniform granular materials with grain sizes roughly between 0.1 mm and 1 mm or potentially for well-graded materials that contain a significant fraction of particles within this range, as long as a representative specimen can be created.

## Application of consolidation models

### Method 1 [15]

The analytical solution developed by Jaeger and Carslaw [13] and further explored by Major and Washington [15] was approximated to determine a temporally variable, depth-averaged coefficient of consolidation. The method was applied to dissipation of the entire layer from the time of peak excess pore pressures until hydrostatic pressures were reached. The analytical solution is given by Eq. 3. A first order approximation was used.

3 $$\begin{array}{*{20}c} {P_{*} = 8P_{{*_{o} }} \mathop \sum \limits_{n = 0}^{\infty } \frac{1}{{\left( {2n + 1} \right)^{2} \pi^{2} }}\cos \left( {\lambda_{n} z} \right){\text{e}}^{{ - \lambda_{n}^{2} c_{v} t}} } \\ \end{array}$$

where *z* is the elevation, *n* is the equation order, *t* is the time since dissipation began, *c*_*v*_ is the coefficient of consolidation, P_*o_ is the initial excess pore pressure at the base elevation, z = 0, and *λ*_*n*_ is an eigenvalue given by Eq. 4.

4 $$\begin{array}{*{20}c} {\lambda_{n} = \frac{{\left( {2n + 1} \right)\pi }}{2H}} \\ \end{array}$$

where *H* is the height of the sample.

**Fig. 8:**
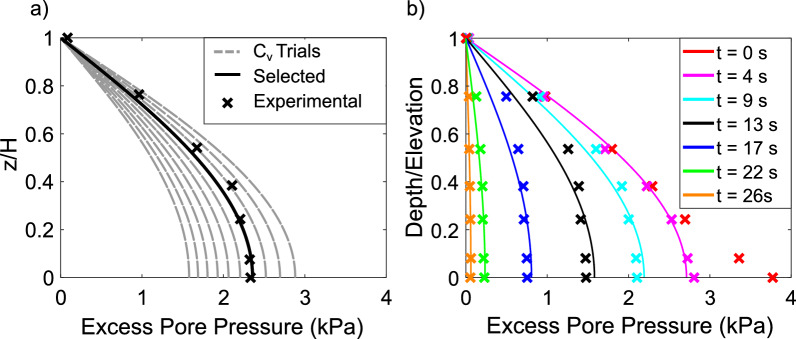
**a** The coefficient of consolidation was determined iteratively using method 1 for material B. A range of coefficients of consolidation were trialled, with the one that produced the least error between isochrones and experimental data being selected; **b** Excess pore pressure isochrones calculated using the determined coefficients of consolidation were plotted alongside experimental results, marked by X’s, at various times during dissipation

Excess pore pressure isochrones calculated from Eqs. 3 and 4 were fitted to experimental results at each time step. The coefficient of consolidation that produced the isochrone with the least error at each time step was selected. Error was calculated as the average absolute difference between each experimental and estimated data points. An example of the selection process is shown in Fig. 8a.

Experimental pore pressure dissipation results along with the corresponding fitted isochrones are shown in Fig. 8b. The selected isochrones match the data well and provide an estimation for the evolution with time of a depth averaged coefficient of consolidation.

### Method 2 [3]

A second analytical solution, method 2, was applied to estimate a depth-averaged coefficient of consolidation. This analytical solution uses a Fourier series approximation given by Eq. 5 [3]:

5 $$\begin{array}{*{20}c} {\overline{u}\left( {z,t} \right) = \mathop \sum \limits_{n = 1}^{\infty } \frac{{8\gamma^{\prime } H}}{{n^{2} \pi^{2} }}\sin \left( {\frac{n\pi z}{{2H}}} \right)\sin \left( {\frac{n\pi }{2}} \right)\exp \left( { - c_{v} \left( {\frac{n\pi }{{2H}}} \right)^{2} t} \right)} \\ \end{array}$$

where $\gamma ^{\prime}$ is the saturated unit weight.

Though two Fourier terms provided the best fit for the data, one term was observed to be sufficient in terms of matching the data well, while allowing for simpler back-calculation of parameters. Three terms over-fitted the observed excess pore pressure dissipation curves. To determine the coefficient of consolidation, the natural logarithm of a first approximation of Eq. 5 was taken, producing Eq. 6.

6 $$\begin{array}{*{20}c} {\ln \left( {\overline{u}} \right) \approx \ln \left( {\frac{{8\gamma^{\prime } H}}{{\pi^{2} }}\sin \left( {\frac{\pi z}{{2H}}} \right)} \right) - \frac{{c_{v} \pi^{2} }}{{4H^{2} }}t} \\ \end{array}$$

This is a linear equation, where the coefficient of consolidation can be calculated from the slope of the natural logarithm of excess pore pressure versus time plot, shown by Eq. 7.

7 $$\begin{array}{*{20}c} {\frac{{\delta \ln \left( {\overline{u}} \right)}}{\delta t} = - \frac{{c_{v} \pi^{2} }}{{4H^{2} }}} \\ \end{array}$$

**Fig. 9:**
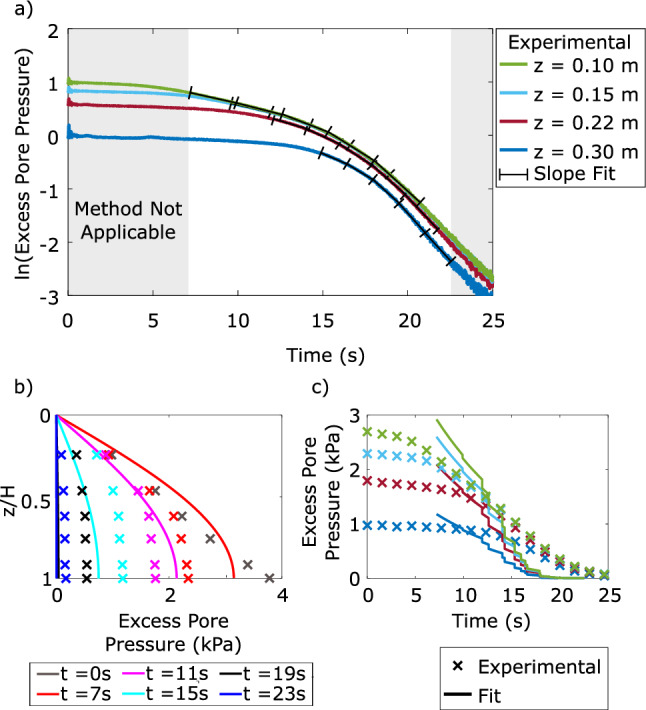
**a** The coefficient of consolidation can be calculated from the slope of the natural logarithm of excess pore pressures versus time plot with method 2 for material B. The model is applicable for excess pore pressure ratios between 0.9 and 0.1, as indicated by the shaded sections, and PPTs at central elevations; **b** Isochrones of excess pore pressure dissipation using calculated coefficients of consolidation; c) Excess pore pressure dissipating over time with fitted results

The natural logarithm of excess pore pressure versus time for four PPTs located at middle elevations of a sample of material B is plotted in Fig. 9. Brennan and Madabhushi [3] recommend this method can only be applied when dissipating pore pressures are between an excess pore pressure ratio of 0.1–0.9. The grey shaded areas of Fig. 9 indicate the area of applicability of the method for each PPT. Moreover, the method was intended for PPTs located at a middle elevation of a sample. The coefficient of consolidation was calculated over five time sections of equal duration, and the calculated coefficients were averaged at each time instance. Each PPT, due to their differing elevations, had excess pore pressure ratios of 0.9 and 0.1 at different times, causing the time increments to not coincide between different PPTs. Fewer time increments were found to not sufficiently capture the nonlinearity of dissipation, while more time increments introduced noise to the final results.

Compared to method 1, isochrones determined using this method did not match the experimental data as well. However, it should be noted that the isochrones were produced using calculated coefficients of consolidation from the excess pore pressure dissipation traces rather than being directly fitted to the isochrone data points, as for the previous method.

### Method 3 [1]

The third method we explore in this paper, method 3, is a numerical solution of the consolidation equation, taking stiffness and hydraulic conductivity as functions of effective stress [1].

While Methods 1 and 2 assume a coefficient of consolidation that is constant with depth but can change with time, Method 3 assumes an effective-stress-dependent coefficient of consolidation, thus allowing both spatial and temporal variation. The relevant differential equation is presented in Eq. 8.

8 $$\begin{array}{*{20}c} {\frac{{\partial \overline{u}}}{{\partial_{t} }} = \frac{{E_{o} }}{{\gamma_{f} }}\left( {\frac{{e_{o} + 1}}{e + 1}} \right)^{2} \left( {\frac{\partial k}{{\partial z_{o} }}\frac{{\partial \overline{u}}}{{\partial z_{o} }} + k\frac{{\partial^{2} \overline{u}}}{{\partial z_{o}^{2} }} - k\frac{{\partial \overline{u}}}{{\partial z_{o} }}\frac{1}{e + 1}\frac{\partial e}{{\partial z_{o} }}} \right)} \\ \end{array}$$

where $\overline{u}$ is excess pore pressure, $z_{o}$ is the Eulerian coordinate for depth, $\gamma_{f}$ is the unit weight of the fluid, $e$ is the current void ratio, $e_{o}$ is the initial void ratio, $E_{o}$ is the one-dimensional stiffness, and $k$ is the hydraulic conductivity.

Similar to the previous methods, the boundary conditions include that no excess pore pressure is allowed at the surface and that no flow can occur at the base of the deposit. Some common assumptions regarding the definition of stiffness and hydraulic conductivity were challenged by this method. One-dimensional stiffness is often defined as a function of effective stress using a power law, which gives zero one-dimensional stiffness at zero effective stress. Noting that one-dimensional stiffness is not shear stiffness, which requires a contact normal force for friction to develop, and that experimental volume change measurements do not justify a complete loss of grain contacts, the assumption made by the method is that a non-zero one-dimensional stiffness can exist at zero effective stress. This assumption allows Eq. 8 to apply throughout a liquefied layer and predict the advancement of the solidification front. This would not be possible with a zero one-dimensional stiffness assumption, which would prevent liquefied soil from ever solidifying [1].

**Fig. 10:**
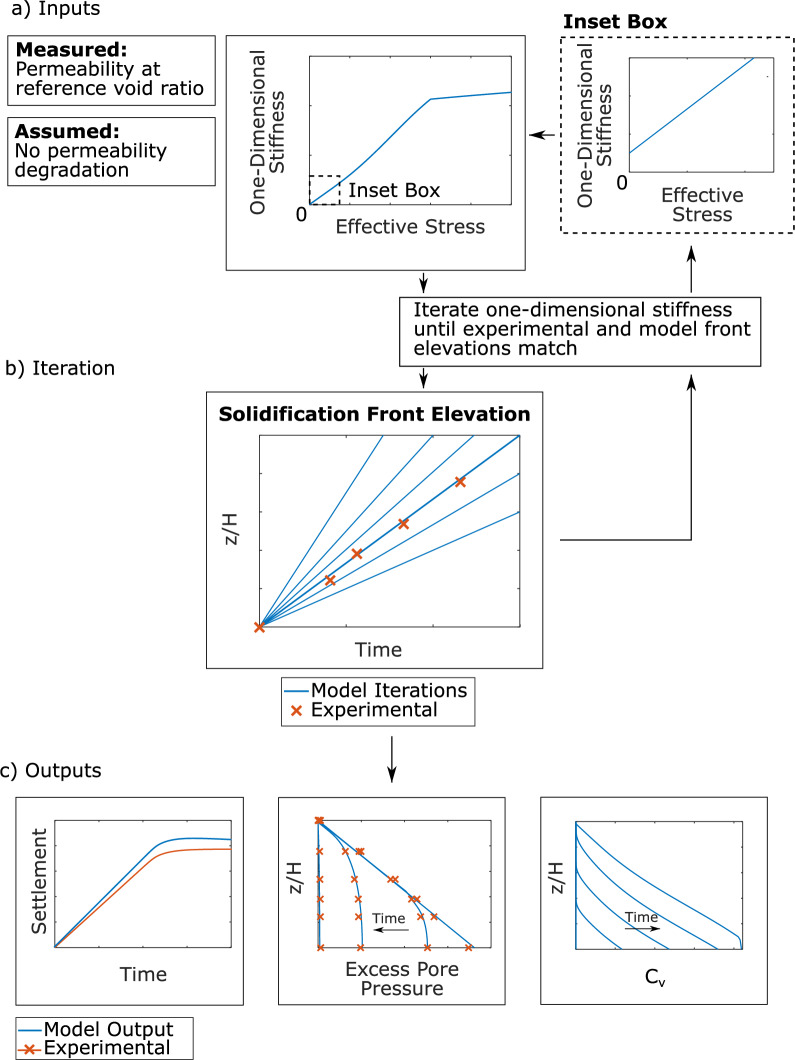
**a** Required model inputs for method 3 include permeability, treated as constant, of the granular material at a reference void ratio. A trial relationship between one-dimensional stiffness and effective stress was implemented, with a non-zero value of one-dimensional stiffness at zero effective stress; **b** The one-dimensional stiffness versus effective stress relationship was iterated until the movement of the consolidation front over time from the model matched experimental results; **c** The model outputs estimated settlement over time, the dissipation of excess pore pressures throughout the depth of the sample, and a temporally and spatially variable coefficient of consolidation

The application process of the numerical solution is shown in Fig. 10. The assumed and measured inputs, iterative process, and outputs are described below.

The hydraulic conductivity of the material was measured from separate lab testing using a permeameter and was assumed here to be constant throughout dissipation, following fluidisation test results that show limited increase for sands with grain diameters similar to materials B and C [10], for which this method was applied. This assumption implies that the coefficient of consolidation, $c_{v}$, has the same effective stress dependency as one-dimensional stiffness, simplifying the process of back-calculation. Materials with finer grains could demonstrate higher increases in hydraulic conductivity with diminishing effective stress. In such cases, this variation could either be taken into account if known, or it could be neglected as a way to simplify back-calculation. In the latter case, the assumed one-dimensional stiffness effective stress dependency will act as a proxy for the effective stress dependency of $c_{v}$.

Capturing one-dimensional stiffness, especially the value of one-dimensional stiffness at zero effective stress, was crucial for modelling the advancement of the solidification front. One-dimensional stiffness was defined as a function of effective stress over three stress ranges. The first stress range, given by Eq. 9, applies for effective stresses less than 0.5 kPa and has to do with the initial values that control the advancement of the solidification front. The third stress range, given by Eq. 11, applies for effective stresses greater than 3 kPa and is a power law, as commonly used for one-dimensional stiffness, which can be determined from oedometer tests. The second stress range, given by Eq. 10, performs a smooth transition between the other two ranges.

9 $$\begin{array}{*{20}c} {E_{o} \left( {{\text{kPa}}} \right) = \frac{{0.841^{2} }}{1.841}\frac{1 + e}{{e^{2} }}\left( {cM1 + aM1\sigma^{\prime }_{v} } \right) , \sigma^{\prime }_{v} \le {\text{d}}M1} \\ \end{array}$$

10 $$\begin{aligned} E_{o} \left( {{\text{kPa}}} \right) = & \frac{{0.841^{2} }}{1.841}\frac{1 + e}{{e^{2} }}*(cM1 + aM1\sigma_{v}^{{^{{\prime}{bM1}} }} + aM2\left( {\sigma_{v}{\prime} } \right)^{{{\text{bM}}2}} \\ & *\frac{1}{2}\left( {1 - \cos \left( {\frac{{\pi \left( {\sigma^{\prime }_{v} - {\text{d}}M1} \right)}}{{{\text{d}}M2 - {\text{d}}M1}}} \right)} \right) , {\text{d}}M1 < \sigma^{\prime }_{v} \le {\text{d}}M2 \\ \end{aligned}$$

11 $$\begin{array}{*{20}c} {E_{o} \left( {{\text{kPa}}} \right) = \frac{{0.841^{2} }}{1.841}\frac{1 + e}{{e^{2} }}\left( {cM1 + aM1*{\text{d}}M2^{{{\text{bM}}1}} + aM2\sigma_{v}^{{\prime^{{{\text{bM}}2}} }} } \right),{\text{d}}M2 < \sigma_{v}^{\prime } } \\ \end{array}$$

where *aM1*, *bM1*, *cM1*, *dM1*, *aM2*, *bM2*, and *dM2* are input parameters. Parameters dM1 and dM2 define the range of application of the above expressions and they were taken as dM1 = 0.5 kPa and dM2 = 3 kPa.

The maximum effective stress for the tests performed was approximately 4 kPa. Therefore, Eq. 11 was beyond the stress range of interest and the parameters that are relevant for it were not investigated here. The parameters of Eq. 9 ($\sigma^{\prime }_{v} \le dM1$) were selected so that the experimentally observed movement of the solidification could be captured. One-dimensional stiffness at greater stresses is important in matching the shape of pore pressure dissipation isochrones, which are controlled by consolidation below the solidification front.

**Fig. 11:**
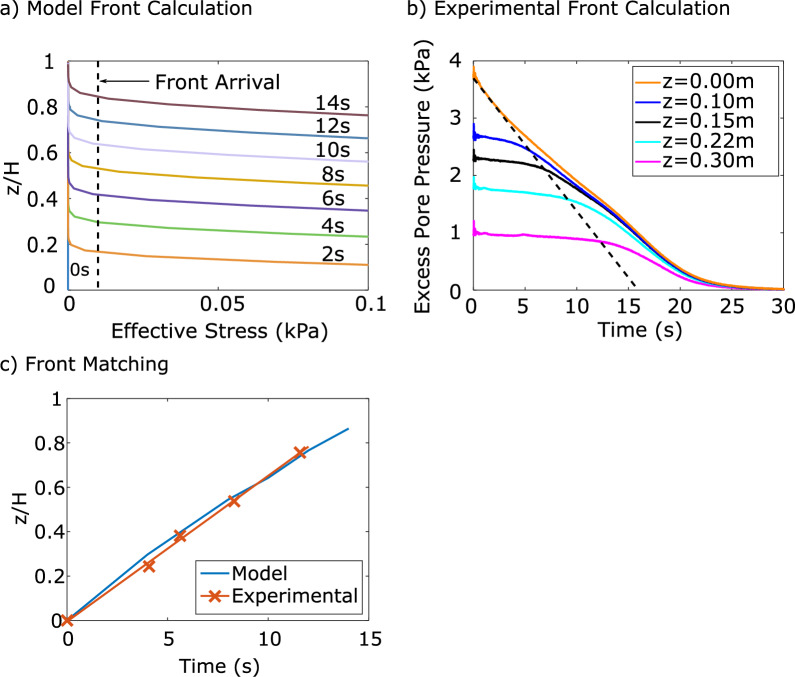
Movement of the consolidation front upwards from the base of the material to the surface can be numerically modelled to match experimental results. **a** In the numerical model, the consolidation front has arrived at an elevation when there is no longer non-zero effective stress, approximated at 0.01 kPa; **b** Excess pore pressure dissipation curves of material B. The consolidation front arrived when constant excess pore pressures had begun to dissipate. A linear approximation was fit to the five points to model front movement; **c** Comparison of the movement of the solidification front as calculated by the numerical model and experimental results

The position of the solidification front was defined as the lowest depth at which effective stress is lower than a threshold of 0.01 kPa, a value selected as approximately zero [1]. The position of the solidification front was tracked upwards through the layer until it reached the surface, as shown by Fig. 11a. The movement of the solidification front from experimental results was determined from excess pore pressure dissipation curves. At each PPT elevation, the time at which excess pore pressures began to decrease from the previously constant values was taken to be the arrival of the solidification front. The solidification front was taken at the base elevation at the initial time of the consolidation analysis. A linear fit was applied to the solidification front movement from the experimental results, as shown by the dashed line in Fig. 11b. Following iterations of one-dimensional stiffness parameter selection, the movement of the solidification front captured from the experiment and computed from the model were well-matched, as shown in Fig. 11c. The selected stiffness parameters are reported in Table 3.

**Table 3:** Calculated stiffness parameters of materials B and C

Material label	Stiffness parameters						
	aM1	bM1	cM1	dM1	aM2	bM2	dM2
B	290	1	2.5	0.5	100	0.6	3
C	350	1	7	0.5	100	0.6	3

**Fig. 12:**
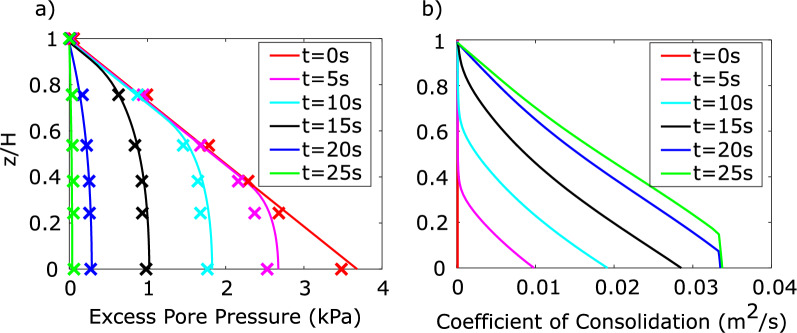
**a** Dissipating excess pore pressures of material B, marked by X’s, fit with numerically calculated isochrones; **b** A spatially and temporally variable coefficient of consolidation was determined

Calculated isochrones of excess pore pressures matched the experimental data well, as shown for time intervals of 5 s in Fig. 12a. The isochrones captured both the advancement of the solidification front as well as the dissipation or pore pressure throughout the entire depth.

The produced temporally and spatially variable estimate of the coefficient of consolidation is shown in Fig. 12b. The coefficient was very close to zero prior to the arrival of the solidification front and increased following its arrival, primarily due to the increase in one-dimensional stiffness.

**Fig. 13:**
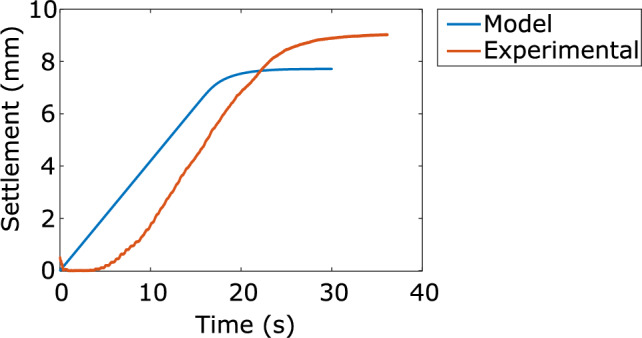
The slope of experimental and numerically modelled surface settlement results match well, and magnitude of settlement estimates were within 15% of each other. Experimental data showed a delay in the initiation of settlement, likely due to experimental error

Surface settlement was also calculated by the model from changes in void ratio as the material consolidates. A comparison of model predicted settlement and experimental settlement results is presented in Fig. 13. The magnitude of predicted settlement matched well with the experimentally observed. A delay in the initiation of the settlement in the experimental results was likely due to experimental error. The plate was jostled by the applied impulse and may have temporarily lost contact with the surface.

### Comparison of consolidation model results

**Fig. 14:**
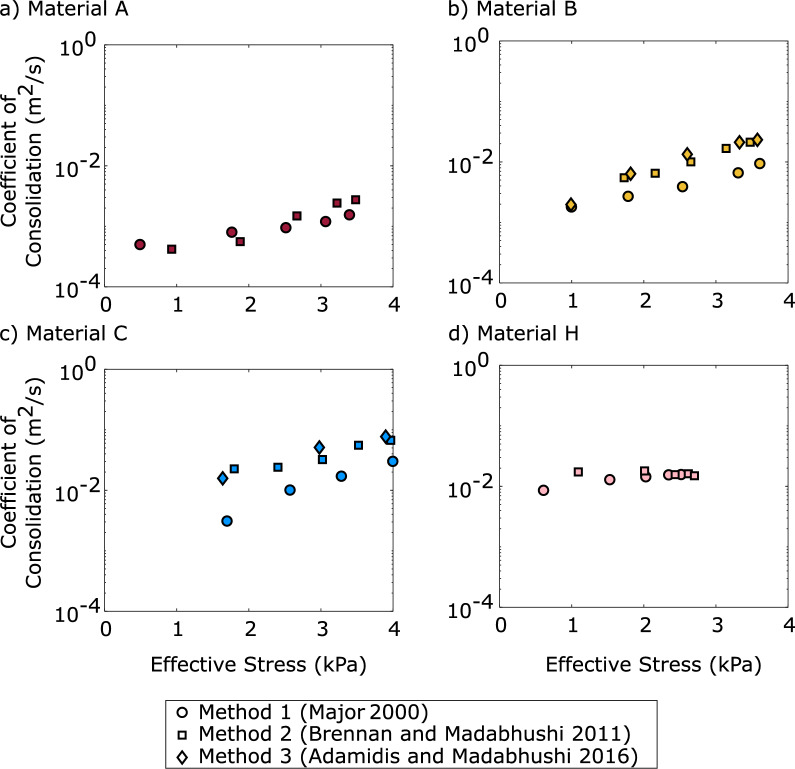
Coefficient of consolidation versus effective stress at the base of the container calculated using Method 1, 2, and 3 for **a** Material A; **b** Material B; **c** Material C; **d** Material H

The coefficient of consolidation was calculated using the previously described three methods. Though Method 3 uses a stress-dependent coefficient of consolidation, the calculated values were depth averaged to allow direct comparison to methods 1 and 2. The coefficients of consolidation for materials A, B, C, and H were plotted against effective stress at the base of the container as calculated by each method in Fig. 14. Method 1 was applied to each material. Method 2 could not be applied to material D as the dissipation time was too short and the data too noisy. Method 3 was applied to materials B and C, where the advancement of the consolidation front was visible in the excess pore pressure dissipation results.

In all cases, the depth-averaged coefficient of consolidation increases during the consolidation process, as effective stresses are regained. Methods 2 and 3 gave similar results for the depth-averaged coefficient of consolidation. Method 1 appears to underpredict the coefficient of consolidation compared to the other two methods, especially as a uniform material becomes more coarse grained. The proposed test seems to be applicable for the well-graded material H, for which increase of the coefficient of consolidation with increasing effective stress was lower than for the poorly graded materials examined.

The coefficient of consolidation varies proportionally with hydraulic conductivity and one-dimensional stiffness according to Eq. 12.

12 $$\begin{array}{*{20}c} {c_{v} = \frac{{k E_{o} }}{{\gamma_{w} }} } \\ \end{array}$$

where *k* is the hydraulic conductivity, $E_{o}$ is the one-dimensional stiffness and $\gamma_{w}$ is the unit weight of water.

As median grain size decreases or a material is becomes more well graded, hydraulic conductivity will decrease. Following Hazen’s commonly used equation, the D_10_ of a material varies with permeability according to Eq. 13.

13 $$\begin{array}{*{20}c} {k = {\text{CD}}_{10}^{2} } \\ \end{array}$$

where *C* is a constant.

Materials with a lower coefficient of consolidation will maintain their excess pore pressures for longer. These coefficients can vary by orders of magnitude for different materials.

**Fig. 15:**
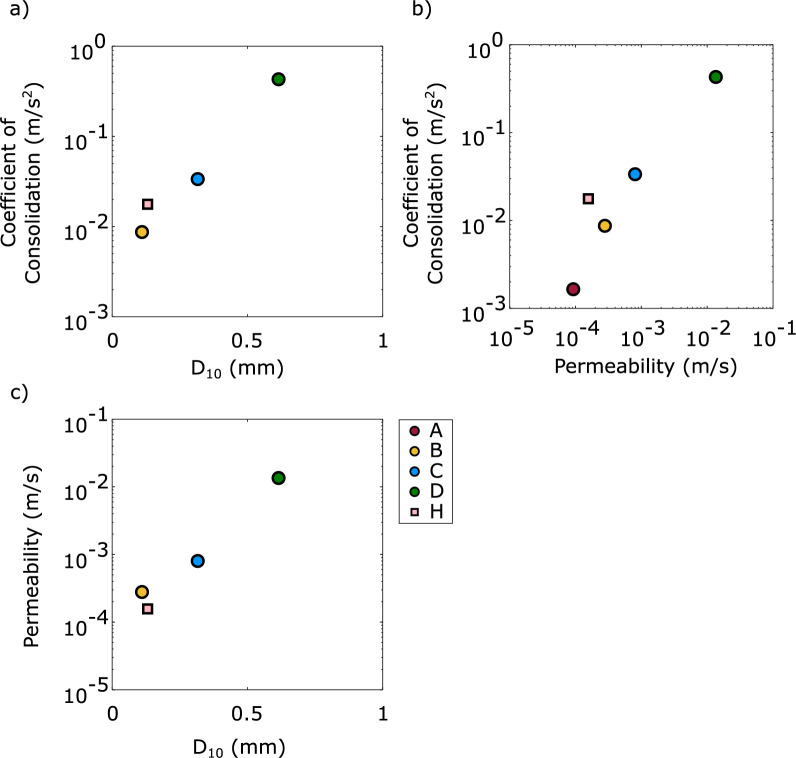
Summary of parameter space explored by the four uniform (**A**–**D**) and one well-graded material (H) exploring the variation in permeability, D_10_, and coefficient of consolidation

The relationships between permeability, D_10_, and fully reconsolidated coefficient of consolidation of the uniform and well-graded materials are presented in Fig. 15. As expected, the coefficient of consolidation increased with increasing D_10_ and permeability.

### Field application

The developed test was performed in laboratory conditions but can easily be deployed in the field. The level of instrumentation required can be based on the desired outputs. A review of each method’s requirements and outputs is presented below and summarised in Table 4.

**Table 4:** Summary of method requirements

	Method 1 [15]	Method 2 [3]	Method 3 [1]
Model type	Analytical	Analytical	Numerical
PPTs required (In Addition to surface PPT)	1 or more	1 or more	At least 3 to capture solidification front movement
Ideal PPT location	At base or throughout layer	Central elevation	Throughout layer
Other instrumentation	–	–	Optional: String potentiometer for settlement validation
Input soil parameters	–	–	Unit weight, hydraulic conductivity
Outputs	Depth-averaged coefficient of consolidation	Depth-averaged coefficient of consolidation	Spatially variable coefficient of consolidation, one-dimensional stiffness, settlement

For an estimation of the depth-averaged coefficient of consolidation, the minimum requirements for the application of method 1 or 2 are a PPT at the surface of the material to measure surface water and at least one additional PPT. This setup requires no additional instrumentation or knowledge of soil properties. The output is a depth-averaged coefficient of consolidation that can be presented as a function of a characteristic effective stress (Fig. 14) so that results can be extrapolated for deeper deposits.

To obtain a more comprehensive investigation into the consolidation properties and estimate one-dimensional stiffness at low stresses, as well as post-liquefaction settlement and a stress-dependent coefficient of consolidation, additional PPTs are required. At least 3 PPTs at various elevations within the sample are needed to track the movement of the solidification front. Though not necessary to predict settlement, a string potentiometer can be added to offer validation for predictions.

The quick consolidation of materials with high permeabilities prevents the use of this test setup. For a layer thickness of 0.35–0.5 m, the test could not be applied to materials coarser than material D as dissipation would occur in less than 0.5 s. The test appears to be applicable for well-graded soils, as long as a non-segregated specimen can be prepared. Due to the short-duration, impulse-nature of our loading, fluidisation conditions throughout the deposit are only imposed for a very short amount of time during the test. As shown in Fig. 7, dissipation of excess pore pressure for material H was quick, meaning that post-fluidisation upwards flow was not maintained for a long time. Though some segregation is inevitable, our back-calculations, which are made on the assumption of a continuum for which the same coefficient of consolidation as a function of effective stress applies (method 3), yielded satisfactory results, indicating that the effects of segregation were limited.

## Conclusions

A series of small-scale tests were conducted in a laboratory setting to develop a low-cost method to easily determine the consolidation parameters of liquified materials. The only requirements for this method were an LLDPE bin, a source of water, a data acquisition system with a sample rate of at least 100 Hz, and at least two pore pressure transducers (PPTs). An impulse is needed to cause initial liquefaction, which in the field can come from a kick to the bucket. The output using analytical models is a depth averaged coefficient of consolidation, which can be expressed as a function of a characteristic effective stress and extrapolated for deeper deposits. A more advanced version of the test requires two additional PPTs and allows the estimation of the actual effective stresses-dependent coefficient of consolidation, and through it the prediction of post-liquefaction settlement. Settlement predictions can be verified with test results if a settlement measurement system, such as a string potentiometer, is added. A good prediction of observed settlement was shown to be possible with the proposed method.

The proposed test provides an opportunity to study post-liquefaction reconsolidation at low stresses. The low stresses at which this test is conducted provides unique insight into this stress range. The relationship between one-dimensional stiffness and effective stress, especially close to zero effective stress, is crucial for capturing the advancement of the solidification front observed in experimental results.

This test is inexpensive, quick, and field-deployable. It can be used to quickly estimate the consolidation properties of liquefiable materials. It performs best for uniform, well-graded materials with dissipation times of at least 0.5 s, for a layer thickness of 0.3–0.5 m. It shows potential when used for well-graded materials, as long as a non-segregated specimen can be prepared. Here, submerged placement was used to prepare a well-graded specimen, which limited segregation but is prohibitive for materials with fines content. Future refinement of the method can target well-graded materials and larger effective stresses.

The test can in the future be adapted for use in the field. It can be used to investigate tailings, debris flows, and sand deposits. It eliminates environmental concerns regarding the transportation and disposal of large volumes of material, especially tailings, in a laboratory setting. Additionally, a field test allows the collection of potentially perishable data and enables tests to be conducted at various locations at low costs.

## Data Availability

Data generated or analysed during this study is available at 10.5683/SP3/EECSU9.

## References

[1] Adamidis O, Madabhushi GSP (2016) Post-liquefaction reconsolidation of sand. Proc Royal Soc A: Math, Phys Eng Sci 472(2186):20150745. 10.1098/rspa.2015.0745 10.1098/rspa.2015.0745 PMC4841662 27118898

[2] Bowman ET, and Sanvitale N (2009). The role of particle size in the flow behaviour of saturated granular materials. Proceedings of the 17th International Conference on soil mechanics and geotechnical engineering: the academia and practice of geotechnical engineering, 1, pp 470–473. 10.3233/978-1-60750-031-5-470

[3] Brennan AJ, Madabhushi SPG (2011) Measurement of coefficient of consolidation during reconsolidation of liquefied sand. Geotech Test J 34(2):139–146. 10.1520/GTJ102914

[4] Collins BD, Reid ME (2019) Enhanced landslide mobility by basal liquefaction: the 2014 State route 530 (Oso), Washington, landslide. GSA Bull. 10.1130/b35146.1

[5] Dobry R (1989) Some basic aspects of soil liquefaction during earthquakes. Ann N Y Acad Sci 558(1):172–182. 10.1111/j.1749-6632.1989.tb22567.x

[6] Dobry R, Alvarez L (1967) Seismic failures of chilean tailings dams. J Soil Mech Found Division 93(6):237–260. 10.1061/JSFEAQ.0001054

[7] Fourie AB, Blight GE, Papageorgiou G (2001) Static liquefaction as a possible explanation for the Merriespruit tailings dam failure. Can Geotech J 38:707–719. 10.1139/cgj-38-4-707

[8] Gohl WB, Jefferies MG, Howies JA, Diggle D (2000) Explosive compaction: Design, implementation and effectiveness. Géotechnique 50(6):657–665. 10.1680/geot.2000.50.6.657

[9] Ha IS, Park YH, Kim MM (2003) Dissipation pattern of excess pore pressure after liquefaction in saturated sand deposits. Transp res rec 1821(1):59–67. 10.3141/1821-07

[10] Haigh SK, Eadington J, Madabhushi SPG (2012) Permeability and stiffness of sands at very low effective stresses. Geotechnique 62(1):69–75. 10.1680/geot.10.P.035

[11] Hamada M, Isoyama R, Wakamatsu K (1996) Liquefaction-induced ground displacement and its related damage to lifeline facilities. Soils Found 36:81–97. 10.3208/SANDF.36.SPECIAL_81

[12] Ishihara K, Yoshimine M (1992) Evaluation of settlements in sand deposits following liquefaction during earthquakes. Soils Found 32(1):173–188. 10.3208/sandf1972.32.173

[13] Jaeger JC, and Carslaw HS (1959). Conduction of heat in solids. *Clarendon P*.

[14] Jana A, Stuedlein AW (2021) Dynamic in situ nonlinear inelastic response of a deep medium dense sand deposit. J Geotech Geoenviron Eng 147(6):1–19. 10.1061/(asce)gt.1943-5606.0002523

[15] Major JJ (2000) Gravity-driven consolidation of granular slurries: implications for debris-flow deposition and deposit characteristics. J sediment res 70(1):64–83

[16] Pierson TC (1981) Dominant particle support mechanisms in debris flows at Mt Thomas, New Zealand, and implications for flow mobility. Sedimentology 28(1):49–60. 10.1111/J.1365-3091.1981.TB01662.X

[17] Rana NM, Ghahramani N, Evans SG, McDougall S, Small A, Take WA (2021) Catastrophic mass flows resulting from tailings impoundment failures. Eng Geol 292:106262. 10.1016/J.ENGGEO.2021.106262

[18] Sassa K, Wang GH (2005) Mechanism of landslide-triggered debris flows: Liquefaction phenomena due to the undrained loading of torrent deposits. Debris-flow Hazards Relat Phenom. 10.1007/3-540-27129-5_5

[19] Terzaghi K (1943) Theoretical soil mechanics. Wiley. 10.1002/9780470172766

[20] Tokimatsu K, Kojima H, Kuwayama S, Abe A, Midorikawa S (1994) LiquefactionInduced Damage to buildings in 1990 Luzon earthquake. J Geotech Eng 120(2):290–307. 10.1061/(ASCE)0733-9410(1994)120:2(290)

[21] Tutfle M, Barstow N (1996) Liquefaction-related ground failure: a case study in the new madrid seismic zone, central united states. Bull Seismol Soc Am 86(3):636–645

[22] Wang G, Sassa K (2003) Pore-pressure generation and movement of rainfall-induced landslides: effects of grain size and fine-particle content. Eng Geol 69(1–2):109–125. 10.1016/S0013-7952(02)00268-5

